# Parental Expressed Emotion Criticism Relates to Everyday Perceptions of Social Threat in Adolescents with Varying Suicidal Thoughts and Behaviors

**DOI:** 10.1007/s10802-025-01416-9

**Published:** 2026-02-16

**Authors:** Kiera M. James, Julianne M. Griffith, Caroline Oppenheimer, Lori N. Scott, Cecile D. Ladouceur, Jennifer S. Silk

**Affiliations:** 1 Department of Psychology, University of Pittsburgh, Pittsburgh, PA USA; 2 Department of Psychiatry, University of Pittsburgh, Pittsburgh, PA USA; 3 Research Triangle Institute International, Durham, NC USA

**Keywords:** Parental criticism, Social threat, Social reward, Ecological momentary assessment, Suicide, Adolescence

## Abstract

Negative parenting behaviors, such as parental criticism, may shape how youth perceive and respond to their day-to-day social experiences, with potential ramifications for social connectedness – a key protective factor for suicidal thoughts and behaviors. The goal of this study was therefore to examine relations between parental expressed emotion criticism and adolescents’ perceptions of social threat and reward during day-to-day peer interactions. Informed by emotion socialization, stress sensitization, and information processing frameworks, we predicted that adolescents with a critical parent would report (i) greater perceptions of social threat and (ii) lower perceptions of social reward during day-to-day peer interactions than adolescents without a critical parent. Participants were 99 adolescents aged 12–17 and their parents recruited from the community. Adolescents were assigned female at birth and 68.3% were at heightened risk for suicidal thoughts and behaviors. Parents completed the Five Minute Speech Sample to assess levels of parental expressed emotion criticism. Adolescents completed baseline questionnaires and a 10-day ecological momentary assessment protocol. Multilevel models tested associations between levels of parental expressed emotion criticism and adolescents’ perceptions of their day-to-day peer interactions. Adolescents with parents high in expressed emotion criticism perceived higher levels of social threat, but not lower social reward, during their real-world peer interactions. Results suggest that adolescents exposed to parental expressed emotion criticism perceive more rejection, exclusion, and peer judgment during their daily interactions with peers, which may have important implications for future suicidal thoughts and behaviors.

**Supplementary Information:**

The online version contains supplementary material available at 10.1007/s10802-025-01416-9.

Adolescence is a critical developmental period characterized by social and affective changes that are driven by neurodevelopment and shaped by socio-contextual and environmental influences. Specifically, adolescents experience maturational changes in neural networks implicated in social processing, responses to social threat/reward, and cognitive control (Casey et al., 2008; Gunnar et al., 2009; Paus et al., 2008; Rudolph, 2014). They nurture greater emotional intimacy in peer relationships and develop heightened awareness of societal expectations, which increases the salience of positive and negative feedback from peers (Blakemore & Mills, 2014; Somerville, 2013), particularly for girls (Rose & Rudolph, 2006). While such changes contribute to socio-affective learning during this key developmental period, adolescents, and especially adolescent girls, also report an increase in interpersonal stressors (Kowalski et al., 2014; Nesi et al., 2019) and demonstrate greater reactivity to those stressors (Guyer et al., 2009, 2012; Leadbeater et al., 1995; Rose & Rudolph, 2006; Weinstein et al., 2006). This heightened sensitivity to social feedback may play an essential role in helping adolescents learn to navigate complex social environments and nuanced social interactions that occur both in-person and online. However, it may also contribute to adolescents’ vulnerability for suicidal thoughts and behaviors (STBs), which have increased dramatically in the last decade among adolescent girls (Centers for Disease Control and Prevention, 2024) and are linked to social threat and reward (e.g., Arango et al., 2019; Cheek et al., 2020; Miller et al., 2024; Mitchell et al., 2018; Oppenheimer et al., 2020).

Despite the increased importance and influence of peer relationships during adolescence, parenting behaviors begin to shape youths’ understanding, experience, and expression of emotion early in childhood (for a review, see Eisenberg et al., 1998) and continue to play an important role in socio-emotional development during adolescence (for reviews, see Baumrind, 1991; Yap et al., 2014). Specifically, from an early age, the ways in which a parent reacts to their child’s emotional reactions socializes that child’s emotion-related reactions (Eisenberg et al., 1998; Halberstadt & Eaton, 2002). For example, Gottman and colleagues (1996) proposed that warm and responsive parenting helps youth learn to regulate their emotions in response to stress. Consistently, youth who report higher perceptions of parental warmth exhibit less neural sensitivity to parental criticism (Butterfield et al., 2021). Additionally, youth whose parents exhibit positive parenting behaviors in the context of stress report less negative emotional reactivity in response to negative peer interactions in daily life (Oppenheimer et al., 2016). These parenting behaviors are associated with reduced risk for negative outcomes broadly, including STBs (Diamond et al., 2022; Wyatt et al., 2015).

Conversely, Gottman and colleagues (1997) also theorized that dismissive and harsh parenting behaviors (e.g., criticism, belittlement) negatively impact a child’s capacity to regulate their emotions as well as their attention in social interactions. Indeed, research shows that negative parenting behaviors, including parental overcontrol, rejection, and criticism, have been linked to problems with socio-emotional functioning (e.g., peer victimization, bullying behaviors; for a review, see Chu & Chen, 2024) as well as STBs (Aiken et al., 2019; Campos et al., 2013; Wedig & Nock, 2007). Prior research also shows that greater maternal negative affect is associated with decreases in adolescents’ responses to social reward (Tan et al., 2014). Much of this research on negative parenting behaviors focuses specifically on how parental expressed emotion criticism (EE-Criticism) – one index of parental criticism – relates to socio-affective functioning and psychiatric risk in youth (Aupperle et al., 2016; James et al., 2018, 2023; James & Gibb, 2019; Silk et al., 2017; Wedig & Nock, 2007). Parental EE-Criticism captures the extent to which a parent expresses criticism, disapproval, or hostility toward their child (Hooley, 1985, 2007). Studies that employ measures of EE-Criticism show that youth who exhibit heightened neural sensitivity to parental criticism report less happiness during social situations in daily life (James et al., 2023) and were more likely to meet criteria for a current episode of major depressive disorder (MDD; Silk et al., 2017). They also link exposure to high levels of EE-Criticism with blunted neural responses to facial displays of emotion (James et al., 2018), as well as STBs and non-suicidal self-injury (NSSI; James & Gibb, 2019; Wedig & Nock, 2007). Nonetheless, research investigating how exposure to parental criticism during adolescence relates specifically to adolescents’ perceptions of social threat and reward during real-world peer interactions is needed to begin to develop a mechanistic understanding of how parental criticism increases risk for STBs during adolescence. Specifically, to the extent that perceptions of social threat and reward during peer interactions impact adolescents’ day-to-day social connectedness, parental criticism may confer risk for STBs during adolescence through its effect on adolescents’ experience of real-world peer interactions.

Beyond emotion socialization processes, complementary frameworks such as stress sensitization and affect-biased information processing models also help conceptualize the effects of parental criticism on adolescent socio-affective functioning. For example, although originally developed to explain recurrence of affective disorders, stress sensitization models have since been extended to explain how early adverse experiences confer heightened reactivity to later social stressors, with implications for a broad range of psychopathology. Specifically, theorists of the stress sensitization model propose that individuals develop increased sensitivity to stress over time through experiencing episodes of affective disorders and exposure to external stressors. This increased stress sensitivity then leads to a progressively lower threshold of stress required to trigger successive affective episodes (Harkness et al., 2015; Monroe & Harkness, 2005). Research supporting this theoretical model shows that childhood exposure to stress sensitizes youth to subsequent stressors, which then increases risk for a wide range of internalizing and externalizing psychopathology (for a review, see Stroud, 2020).

Complementing this theoretical framework, still other researchers propose that adverse childhood experiences influence the development of experience-specific biases in attention, interpretation, and memory for affectively-salient information (see Cicchetti et al., 2000; Pollak, 2003; Rose & Abramson, 1992). As such, repeated exposure to, for example, threatening emotional expressions, such as anger (e.g., in the context of parental criticism), may contribute to the development of hypervigilance for these cues so that future experiences of threat (e.g., criticism) can be avoided (Cicchetti et al., 2000; Pollak, 2003). This pattern of biased threat processing could manifest as increased sensitivity in recognizing facial cues of threat or as greater attention to threat-relevant cues or information (Gulley et al., 2014; Pollak & Tolley-Schell, 2003).

Integrating these theoretical models and prior research, we propose that adolescent girls exposed to high levels of parental criticism may be especially attuned to future social stressors (e.g., peer conflict, victimization), such that these adolescents develop increased sensitivity to detecting cues of social threat (e.g., a critical, unkind, or sarcastic tone of voice, hostile language, or angry facial expression) during peer interactions or are more prone to interpret social cues (e.g., a neutral verbal response or facial expression or being left “on read” in a messaging application) as threatening (e.g., a potential signal of peer rejection). Further, to the extent that these adolescents develop biased processing of threat-relevant social cues, they may overlook or disregard cues signaling social reward. Nonetheless, as stated above, to our knowledge, no studies have examined the effects of exposure to parental criticism on adolescents’ real-world socio-affective functioning, which may have important implications for the development of more targeted just-in-time prevention and intervention to reduce STBs among adolescent girls. For example, ecological momentary intervention, which leverages mobile technology to provide intervention to throughout daily life (Heron & Smyth, 2010), could be used to help adolescents more objectively evaluate their real-world social interactions and redirect their attention from (potential) signals of social threat to those of reward in an effort to increase social connectedness.

Thus, the current study leveraged behavioral observation, questionnaire, and ecological momentary assessment (EMA) methods in a multi-modal effort to understand how adolescent girls with and without a critical parent perceive social threat and reward during interactions with their peers in daily life. Although we present several possible frameworks for understanding how exposure to parental criticism might relate to adolescents’ perceptions of social threat or reward during their real-world peer interactions above (i.e., emotion socialization, stress sensitization, and affect-biases information processing), the current study was not designed to test a mechanistic model. Rather, the primary goal of this study was to understand the extent to which exposure to parental criticism is associated with adolescents’ real-world socio-affective functioning (i.e., perceptions of threat and reward during peer interactions) to lay the foundation for future mechanistic research. Ecological momentary assessment, which employs signaling devices (e.g., cell phones) to collect real-time data on emotion and behavior in the natural environment, was used to provide an ecologically valid and reliable measure of adolescents’ perceptions of their real-world peer interactions (Hormuth, 1986; Larson & Csikszentmihalyi, 1983; Stone et al., 1999). This approach is ideal for increasing the ecological validity of research on socio-affective processes and experiences, like adolescents’ perceptions of peer interactions, that can fluctuate rapidly within-person and over time. Assessing adolescents’ experiences of their peer interactions in real-time (versus relying on their retrospective report of their experiences of peer interactions over a long period of time) may be important given the frequency with which youth interact with their peers across in-person and online environments. In particular, measuring youths’ experiences of interpersonal stressors in daily life may be especially important in light of ample research highlighting the role of interpersonal stressors in increasing risk, including risk for STBs (Campos et al., 2013; Giletta et al., 2015; Liu & Miller, 2014; Mitchell et al., 2018; Oppenheimer et al., 2020). Peer rejection and victimization, specifically, have been consistently linked to STBs in cross-sectional and longitudinal research, and these relations appear to be strongest for girls (King & Merchant, 2008; Massing-Schaffer et al., 2022; Heilbron & Prinstein, 2010), suggesting that the way in which girls experience social threat may increase their vulnerability for STBs.

As such, in the current study, we first examined the extent to which adolescent girls with and without a critical parent perceive day-to-day interactions with their peers as socially threatening. Mirroring these analyses, we then examined the extent to which adolescent girls with and without a critical parent perceive day-to-day interactions with their peers as socially rewarding. We hypothesized that adolescents with a critical parent would report (i) greater perceptions of social threat and (ii) lower perceptions of social reward during day-to-day social interactions with peers than adolescents without a critical parent. Finally, given that adolescents with depression (Calleja & Rapee, 2020; Platt et al., 2017) and anxiety (Calleja & Rapee, 2020; Dapprich et al., 2023) are likely to interpret social interactions as threatening and that participants in our sample were recruited based on their past-year STBs, we conducted follow-up tests of robustness in which we statistically adjusted for adolescents’ symptoms of anxiety and depression, as well as baseline levels of suicidal ideation.

## Method

### Participants

Participants included 99 adolescents assigned female at birth ages 12–17 (*M* = 15.31, *SD* = 1.51; 68.3% at elevated risk for suicidal thoughts and behaviors) and a participating parent (*M* = 46.10, *SD* = 5.67; 96% mothers).^1^ With respect to gender, 77.8% identified as girls, 11.1% as nonbinary, 4.0% as transgender, and 6.1% as another gender minority.^2^ With respect to race, 1% of youth identified as Asian, 4% as Biracial, 7% as Black, 86% as White, and 1% as another racial identity. 3% of youth identified as Hispanic/Latine. Median family income was $90,000-$100,000. Demographic and clinical characteristics are presented in Table 1.

**Table 1:** Descriptive statistics and bivariate correlations describing primary variables of interest

	*Potential Range*	M(SD) Overall	M(SD) High Parental Criticism (*n* = 53)	M(SD) Low Parental Criticism (*n* = 46)	t(df)	*p*	Cohen’s d	1.	2.	3.	4.	5.
1. EMA Social Threat	0–100	29.15 (20.17)	20.67 (2.84)	17.82 (2.63)	−2.97 (97)	0.004	− 0.60	---				
2. EMA Social Reward	0–100	63.15 (16.37)	15.28 (2.10)	17.11 (2.52)	1.89 (97)	0.061	0.38	**− 0.37**	---			
3. SIQ-Jr	0–90	20.17 (20.62)	24.29 (20.99)	15.19 (19.24)	−2.18 (93)	0.031	− 0.45	**0.48**	**− 0.38**	---		
4. Age	12–18	15.31 (1.51)	15.38 (1.49)	15.23 (1.54)	− 0.49 (97)	0.625	− 0.10	− 0.01	− 0.08	0.06	---	
5. MFQ	0–66	22.56 (15.09)	25.78 (13.74)	18.63 (15.89)	−2.30 (89)	0.024	− 0.49	**0.58**	**− 0.31**	**0.81**	0.11	---
6. SCARED	0–88	40.04 (20.82)	42.21 (19.21)	37.43 (22.55)	−1.23 (95)	0.263	− 0.23	**0.50**	**− 0.26**	**0.54**	0.05	**0.74**

For descriptive purposes, person-mean social threat and social reward ratings were used for calculating grand-mean values and bivariate correlations estimates. Suicidal ideation measures were missing for *n* = 4 participants, depressive symptom measures were missing for *n* = 8 participants, and anxiety symptom measures were missing for *n* = 2 participants. Bolded values are significantly different from zero at *p* <.05. *EMA* ecological momentary assessment, *SIQ* suicidal ideation as measured using the suicidal ideation questionnaire, *MFQ* depressive symptoms as measured using the mood and feelings questionnaire (suicidality items removed); *SCARED* anxiety symptoms as assessed using the Screen for Childhood Anxiety and Related Disorders

Data were collected in association with a larger ongoing study (see Teen SCREEN Study; https://osf.io/f6d7w). Inclusion criteria for this study include female sex assigned at birth and access to personal smartphone device compatible with passive sensing software. Participants were stratified based on risk for suicidal thoughts and behaviors, with “high-risk” youth operationalized as youth endorsing one or more of the following: (a) recurrent suicidal ideation (including passive death wish) within the past six weeks, (b) five or more episodes of NSSI in past year, or (c) suicide attempt in past year. Exclusion criteria include being unable to read and complete questionnaires in English, the presence of magnetic resonance imaging (MRI) contraindications, pregnancy, a lifetime presence of neurological disorder and/or Diagnostic and Statistical Manual of Mental Illnesses Fifth Edition (DSM-5) psychotic disorder or autism spectrum disorder, a severe current substance use disorder, or a history of serious head injury or neurological anomaly. The present study focuses on behavioral observation data obtained via the Five Minute Speech Sample (FMSS), questionnaire data obtained at the baseline assessment, and EMA data collected across a 10-day assessment period. Adolescents completed three 10-day bursts of EMA during a three month period. The current study utilizes data from the first 10-day EMA period for which full data is available for all participants. All participants who completed both the FMSS and first 10-days of EMA were included for the purposes of the present analyses.

Participants were recruited from the general community through social media (e.g., Instagram); print advertisements (e.g., brochures, flyers, postcards) distributed by mail, on buses, and in community centers; and study representatives who tabled at local events. Participants were also recruited from local outpatient clinics through flyers and targeted mailings to families in the University of Pittsburgh research registry. Participants completed the FMSS at the beginning of a laboratory visit involving a series of parent-teen dyadic interaction tasks for which parents were paid $15. Adolescent participants were paid up to $80 for completing EMA questionnaires (including bonuses for compliance). Payment was determined by the percentage of phone questionnaires completed.

### Measures

#### Ecological Momentary Assessment of Perceived Social Threat and Reward

Girls’ perceptions of social threat and reward during interactions with peers in daily life were assessed using EMA. Girls were prompted three times per day to respond to a brief survey on their personal smartphone devices over the course of the EMA data collection procedure (total possible prompts = 30). On each survey, girls were asked to think about their interactions with other teens their age that occurred since their last completed survey and to rate their perception of social threat or reward during those interactions using a 0 (not at all) to 100 (extremely) visual analog scale. Girls were asked to rate their perceptions of social threat and reward during both in person and online peer interactions separately. Items assessing social threat and reward were adapted from a previously published protocol from our group (Sequeira, Silk, et al., 2021) and derived based on theories and empirical work describing social threat and reward (Baumeister & Leary, 1995; Dickerson, 2008; Dickerson & Kemeny, 2004; Foulkes & Blakemore, 2016). Specifically, for both types of interactions, to capture perception of social threat, girls rated how much they felt (1) disliked, criticized, or rejected, (2) ignored, lonely, or excluded, and (3) embarrassed or worried about what someone thought of [them]. All three items were correlated with one another at the within-person level (*r*s = 0.37–0.60.37.60, all *p*s < 0.001) and between-persons levels (*r*s = 0.83–0.95.83.95, all *p*s < 0.001)^3^. Multilevel reliability estimates calculated using the ‘multilevelTools’ package in R (Wiley, 2020) indicated adequate internal consistency at both the within- (ω =.73) and between-persons (ω =.94) levels. Girls’ ratings on each of these items were averaged together, and scores were averaged across in-person and digital contexts to create a social threat score that was used in analyses. To capture perception of social reward, girls rated how much they felt 1) close, connected, and comfortable with people, 2) accepted and part of the group, and 3) having fun with people. The three social reward items also significantly correlated at the within- (*r*s =.73-.76, all *p*s <.001) and between-persons levels (*r*s =.95-.98, all *p*s <.001). Internal consistency was good at the within- (ω =.89) and between-persons levels (ω =.99). As with the perceived social threat items, girls’ ratings on each of these items were averaged together and across contexts to create a social reward score at each time point.

#### Expressed Emotion – Criticism

Parental expressed emotion criticism (EE-criticism) was assessed using the Five Minute Speech Sample (FMSS; Magaña et al., 1986). To administer the FMSS, the parent is asked to speak for five uninterrupted minutes about their daughter and how they get along with their daughter. The response is audiotaped and coded by independent raters for levels of EE-criticism. Parents are rated as high on EE–criticism if (1) their initial statement about their daughter is negative (e.g., “Mia is a very difficult person.”), (2) they report a negative relationship (e.g., “Jocelyn and I yell at each other all the time.”), or (3) they report one or more criticisms as defined by the FMSS coding system (e.g., “I don’t like her,” “It makes me mad that she…,” “She drives me crazy”) as defined by the FMSS coding system. Parents are rated as borderline critical if they express dissatisfaction with their daughter not severe enough to be rated as a criticism (e.g., “I’d rather she was not like that,” “It bothers me that she…,”). Responses to the FMSS were assigned values of 2, 1, and 0 to reflect high, borderline high, and low EE–criticism, respectively. Consistent with recommendations that the FMSS, if anything, tends to under-identify individuals with high EE–criticism (Hooley & Parker, 2006) and with previous research using the FMSS (e.g., Gar & Hudson, 2008; James et al., 2017, 2021; James & Gibb, 2019), responses were dichotomized such that parents exhibiting borderline or high EE–criticism were classified as critical (*n* = 53) and parents exhibiting low EE–criticism were classified as not critical (*n* = 46). A number of studies have supported the reliability and validity of the FMSS EE–criticism subscale, including concurrent validity with observer ratings of criticism during actual parent–child interactions (Asarnow et al., 2001; Magaña et al., 1986; Rogosch et al., 2004). In this study, the FMSS was coded by undergraduate-, post-baccalaureate-, and post-doctoral-level individuals who were trained to reliability standards and were blind to the other study variables. All samples were independently coded by two raters; when discrepancies arose, a third rater was consulted, and a consensus rating was reached. Inter-rater reliability was assessed with a subset of 33 speech samples, and the reliability of EE–criticism ratings was good (κ = 0.88).

#### Symptoms

To better characterize the sample and for use in sensitivity analyses, girls’ suicidal ideation, as well as symptoms of depression and anxiety were assessed at baseline. Girls’ suicidal ideation was assessed using the Suicidal Ideation Question – Junior (SIQ-Jr; Reynolds, 1988), which contains 15 items assessing adolescents’ suicidal thoughts over the past month on a seven-point Likert scale, ranging from 0 “*I have never had this thought*” to 6 “*Almost every day*”. Items were summed to create a total score, where a higher score reflects more symptoms (range 0 to 90). The SIQ-Jr demonstrated excellent internal consistency in the current study (ω = 0.97). Depressive symptoms were assessed using girls’ report on the Mood and Feelings Questionnaire (MFQ; Angold et al., 1995), which contains 33 items assessing symptoms over the past *two* weeks on a three-point Likert scale (0 = *not true*, 1 = *sometimes*, 2 = *true).* Consistent with prior research (Hutchinson et al., 2021; Miller et al., 2024), four items assessing suicidality were removed to avoid overlap with the SIQ-Jr, yielding 29 total items. These MFQ items were summed to create a total score, where a higher score reflects more symptoms (range 0 to 58). Anxiety symptoms were assessed using girls’ report on the Screen for Anxiety Related Disorders (SCARED; Birmaher et al., 1997), which contains 44 items assessing anxiety symptoms over the past week on a three-point Likert scale (0 = *not true/hardly ever true*, 1 = *somewhat true/sometimes true*, 2 = *very true/often true*). The 44-item SCARED used in the present work combines the original 41-item SCARED (Birmaher et al., 1997) with the social phobia scale of the SCARED-71 (Bodden et al., 2009). SCARED items were summed to create a total score where a higher score reflects greater symptoms (range 0 to 88). Both the MFQ and SCARED demonstrated excellent internal consistency in the current study (ωs = 0.96 and 0.96, respectively). Means and standard deviations are presented in Table 1.

### Procedure and Ethical Considerations

Before enrollment, parents provided informed consent and girls provided assent to be in the larger, longitudinal study. At baseline, girls completed questionnaires assessing their levels of suicidal ideation and symptoms of anxiety and depression. Parents completed the FMSS. Following these baseline assessments, girls completed 10 days of EMA. Specifically, girls were assisted with accessing a secure WebDataExpress (WDX) web portal designed for the completion of EMA surveys on their personal smartphone devices. Girls were then prompted to respond to brief EMA surveys at three semi-random times (morning, afternoon, evening) each day for 10 days using the WDX portal (*n* = 30 total EMAs). Girls were permitted to select the time at which their morning survey arrived within a 30-minute window. Afternoon surveys were delivered at random times between 3:30 − 6:30pm, and evening surveys were delivered at random times between 6:30 − 9:30pm. Girls had 60 min to complete each survey before it expired. All study procedures were approved by the University of Pittsburgh Institutional Review Board.

### Data Analytic Plan

Hypotheses were tested using a series of multilevel models using restricted likelihood estimation (REML) implemented using the ‘lme4’ package in R (Bates et al., 2015; R Core Team, 2022). Multilevel models including random intercepts representing individual differences in levels of perceived social threat and reward outcomes were used to account for nesting of individual observations within people, and maximize the richness of the data.^4^ To evaluate associations between parental criticism and youth perceptions of social threat and reward, we first tested models in which dichotomous parental criticism scores were entered as predictors of EMA-assessed perceptions of social threat and reward, respectively, averaged across in-person and digital contexts. In instances in which associations were significant, we then conducted sensitivity analyses evaluating associations between parental criticism and perceptions of social threat/reward across in-person and digital contexts, separately. We also conducted sensitivity analyses statistically adjusting for adolescents’ age and EMA completion rate, as well as baseline level of suicidal ideation given that youth were recruited based on their history of STBs, and symptoms of depression and anxiety at baseline, given that adolescents with depression (Calleja & Rapee, 2020; Platt et al., 2017) and anxiety (Calleja & Rapee, 2020; Dapprich et al., 2023) are likely to interpret social interactions as threatening. Continuous level 2 covariates were grand mean-centered prior to analysis. 

## Results

### Preliminary Analyses

**Fig. 1:**
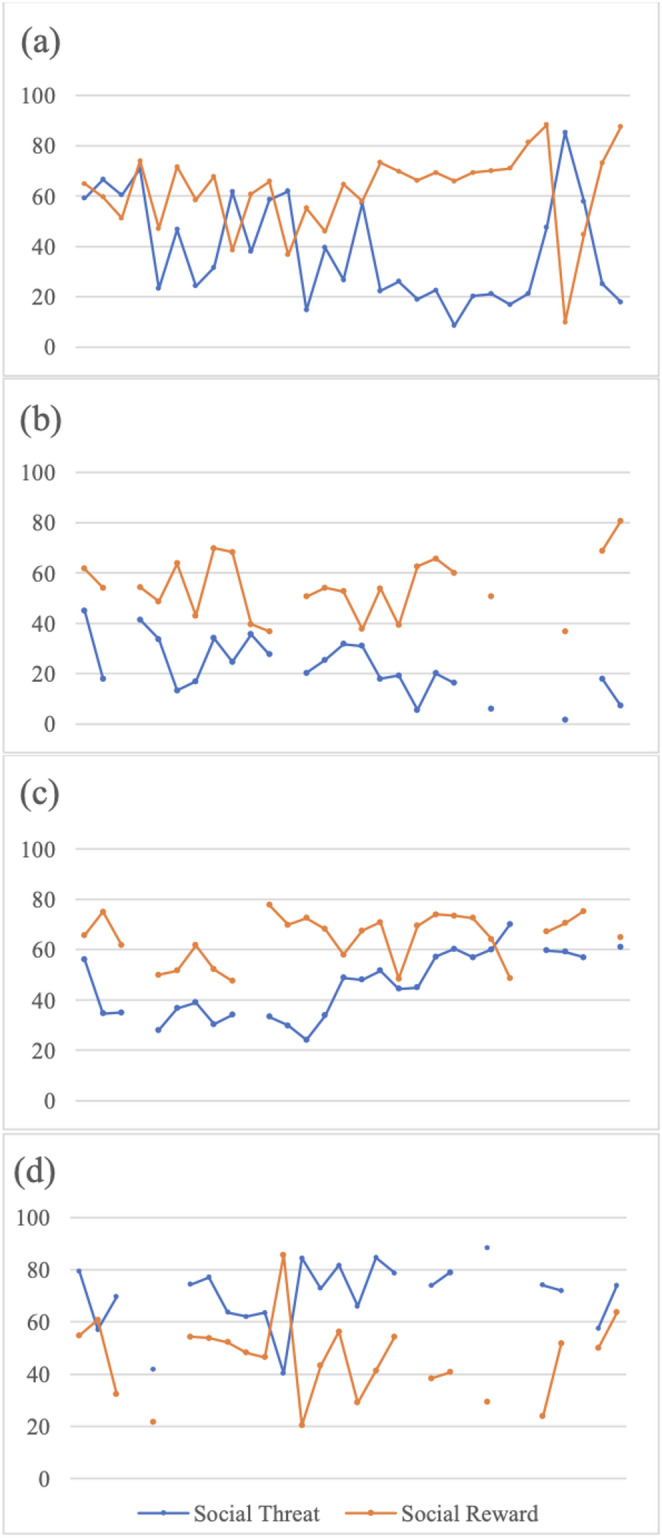
Examples of social threat and reward ratings across the EMA period in low parental EE-criticism **(a-b)** and high parental EE-criticism **(c-d) **groups

EMA survey completion rate was 77% (range = 17–100%). Completion rate was positively correlated with age (*r =*.20, *p =*.048), but not significantly associated with baseline suicidal ideation, parental criticism, or mean perceived social threat or reward ratings (*p*s > 0.05). Descriptive statistics and bivariate correlations characterizing primary variables of interest are reported in Table 1. Parental criticism was positively associated with increased mean perceived social threat ratings and suicidal ideation. Intraclass correlation (ICC) values parsing variance indicated that levels of perceived social threat (ICC = 0.64) and reward (ICC = 0.42) both varied across timepoints within a person, as well as on average, across people. Examples of participant social threat and acceptance ratings over the EMA period are presented in Fig. 1.

### Associations between Parental Criticism and Daily-Life Perceptions of Social Threat and Reward

**Fig. 2:**
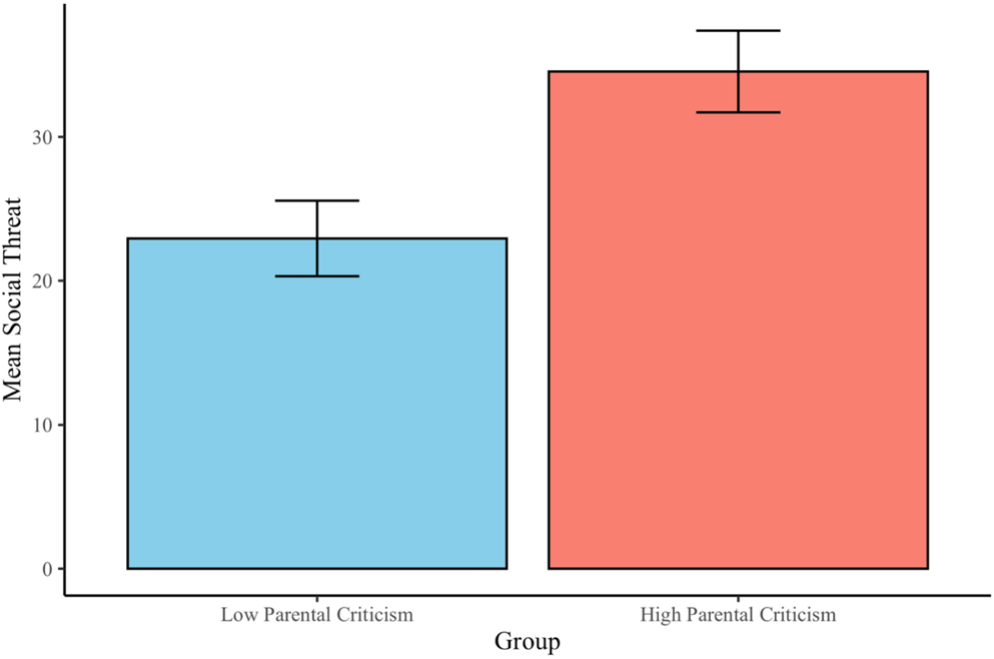
Mean differences in perceived social threat by parental criticism group. For the purposes of visualization, perceived social threat ratings were averaged across EMA per participant. Error bars represent standard error of the mean

Complete results of multilevel models evaluating associations between parental criticism and adolescents’ perceptions of social threat and reward are reported in Table 2. Findings indicate that parental criticism was positively related to adolescents’ perceptions of social threat in daily-life peer interactions (*b =* 11.35, *SE* = 3.89, *p* =.004; see Fig. 2). Results were consistent across both in-person and digital contexts (see Table 2). The effect was maintained in sensitivity analyses restricting the sample to mothers (*b* = 10.75, *SE* = 3.96, *p* =.008), and remained significant at a trend level in a test of robustness in which adolescents’ current symptoms of depression, anxiety, and suicidal ideation were included alongside age and EMA completion rate in the model as covariates (*b* = 7.24, *SE* = 3.80, *p* =.060; see Supplemental Table S1). Parental criticism was not significantly related to adolescents’ perceptions of daily-life social reward (*b*=−6.27, *SE* = 3.28, *p* =.059).^5^^,^^6^

**Table 2:** Results of multilevel models evaluating associations between parental criticism and social Threat/Reward

	b	SE(b)	*p*
Parental Criticism ◊ Perceived Social Threat			
Intercept	23.20	2.85	< 0.001
Parental criticism	11.35	3.89	0.004
Parental Criticism ◊ Perceived In-Person Social Threat			
Intercept	26.07	3.01	< 0.001
Parental criticism	8.47	4.08	0.041
Parental Criticism ◊ Perceived Digital Social Threat			
Intercept	22.37	2.97	< 0.001
Parental criticism	10.47	4.09	0.012
Parental Criticism ◊ Perceived Social Reward			
Intercept	66.55	2.41	< 0.001
Parental criticism	−6.27	3.28	0.059

*b* = unstandardized effect; SE(*b*) = standard error of the unstandardized effect

## Discussion

Results of the current study show that girls with parents high in EE-Criticism perceived more social threat, though not less social reward, during day-to-day peer interactions than girls with low EE-Criticism parents. This pattern of results suggests that the effects of parental criticism may be specific to detection of (and sensitivity to) cues of social threat, rather than girls’ processing of social cues signaling reward from peers. Moreover, that adolescents reported similar levels of perceived social reward regardless of exposure to a critical parent may reflect some resilience within this risk-enriched sample. This pattern may be indicative of the presence of protective processes (e.g., emotion regulation) that enable youth to maintain sensitivity to rewarding aspects of peer interactions, even in the context of heightened social threat sensitivity, and highlights the potential separability of threat- and reward-related socioaffective processes, which may differentially contribute to risk and resilience pathways.

Notably, the relation between parental criticism and perceptions of social threat in real-world peer interactions remained significant at a trend level when statistically adjusting for the influence of adolescents’ symptoms of anxiety and depression as well as their baseline levels of suicidal ideation, suggesting that this effect was at least partially independent of these clinical influences. The present findings underscore an association between exposure to a critical parent and adolescents’ real-world socioaffective functioning, which may, in turn, have implications for adolescents’ risk for STBs. Future longitudinal research linking laboratory-assessed processes, like parental criticism, with real-world behavior and STBs is crucial to understanding trajectories of (and fluctuations in) risk as well as for identifying specific behavioral prevention and intervention targets. This research is also needed to test the precise mechanisms through which exposure to parental criticism relate to day-to-day socioaffective functioning during adolescence.

Interestingly, the specificity of current findings to perceived social threat diverges from the results of peripherally related prior research showing that higher parental negative affect during parent-adolescent interactions relates to adolescents’ neural sensitivity to peer acceptance, but not rejection (Tan et al., 2014). The discrepancy in findings of current and past research may be explained by methodological differences. For instance, parental negative affect and parental criticism, though related, are different constructs, and unlike parental criticism, the expression of negative affect is not inherently relational, even in the context of social interaction. Additionally, the current study examined adolescents’ *perceptions* of social threat and reward during day-to-day peer interactions whereas Tan and colleagues (2014) focused on *neural sensitivity* to simulated experiences of overt peer rejection and acceptance. Finally, whereas Tan and colleagues’ (2014) study was conducted in a sample of adolescent boys and girls, the present study focused exclusively on female adolescents, who experience higher rates of socially threatening experiences and sensitivity to social threat than boys (Ge et al., 1994; Rose & Rudolph, 2006; Rudolph & Hammen, 1999; Simonson et al., 2011). Regardless, the discrepancy in findings between these two studies suggests a need for future research in this area to better understand how negative parenting behaviors relate to adolescents’ socio-affective functioning, including potential transactional or bi-directional effects.

Consistent with a stress sensitization framework (Harkness et al., 2015; Monroe & Harkness, 2005), one possible interpretation of the current results is that parental criticism may function as a salient stressor sensitizing high-risk adolescent girls to other stressors in their daily lives (e.g., during interactions with their peers), such that adolescents exposed to parental criticism perceive higher levels of social threat during real-life peer interactions. Results also offer potential real-world behavioral support for affect-biased information processing models (Cicchetti et al., 2000; Pollak, 2003; Rose & Abramson, 1992). Specifically, the present findings build upon prior research that shows that youth who have experienced physical abuse are more likely to over-attend to cues of social threat (i.e., angry faces; Pollak & Tolley-Schell, 2003) as well as research showing that, even in adulthood, those who report experiences of childhood maltreatment show heightened neural sensitivity to threatening facial expressions (i.e., fearful faces; Sandre et al., 2018). The current findings also align with studies investigating the role of more typical parenting behaviors in the development of experience-specific information processing biases (Gibb et al., 2011; Gulley et al., 2014), which are consistent with emotion socialization frameworks (e.g., Gottman et al., 1997). These studies show that youth exposed to negative parenting styles and/or behaviors (i.e., authoritarian parenting, parental criticism, parental negative affect) show affect-biased attention for threatening facial expressions (i.e., angry faces; Gibb et al., 2011; Gulley et al., 2014).

Consistent with other theoretical frameworks (Bowlby, 1969, 1988), another possible explanation for the current pattern of results is that youth exposed to parental criticism may come to view themselves more negatively, which might lead them to expect peers to view them negatively as well. To this end, in the current study, adolescents’ heightened perception of social threat during peer interactions may reflect the impact of parental criticism on adolescents’ own self-worth. Supporting this explanation, several prior studies highlight associations between negative parenting behaviors, including parental criticism/rejection and self-criticism (Brewin et al., 1996; Campos et al., 2013; Clark & Coker, 2009) with one study suggesting that parental criticism is associated with self-criticism among girls, but not boys (Clark & Coker, 2009). Alternatively (or additionally), and supporting the stress generation hypothesis (Hammen, 1991, 2005), it may be that the adolescents with critical parents, who also reported higher levels STBs and symptoms of depression than the adolescents without critical parents in the current study, generate more interpersonal stress. In this case, these youths’ perceptions of more social threat during peer interaction may reflect their objective experiences (e.g., lower quality relationship and more conflict). Supporting this explanation, research shows that elevated symptoms of depression and STB prospectively predict increases in self-generated stress in youth (for reviews, see Liu & Alloy, 2010; Liu & Spirito, 2019).

Finally, the current results may reflect a transactional or bidirectional association between parental criticism and adolescents’ perceptions of social threat in everyday life. That is, adolescents’ heightened sensitivity to social threat may also shape how their parents (and peers) respond to them, creating and perpetuating cycles of criticism, conflict, and perceived rejection. For example, to the extent that adolescents who are more sensitive to social threat are also more emotionally reactive or socially insecure, one possibility is that these youth elicit more criticism from their parents. It is also possible that certain adolescent behaviors that evoke criticism from parents may similarly frustrate or alienate peers, leading to real social strain that adolescents accurately perceive as threatening. Future longitudinal research is needed to clarify the directionality and transactional nature of these associations.

It is also important to note that the focus of the current study on *exposure* to parental criticism, as determined via behavioral coding using gold-standard assessment methods, complements findings from previous studies that examined socioaffective associations of *sensitivity* to parental criticism (e.g., Butterfield et al., 2021; James et al., 2023). Taken together, these studies suggest that both exposure *and* sensitivity to parental criticism are related to alterations in socio-affective functioning, though future research assessing exposure *and* sensitivity to parental criticism and implications for socio-affective functioning during adolescence is necessary to determine the extent to which these factors relate to socio-affective functioning through overlapping or distinct pathways. It is also worth noting that these findings also align with previous research showing that adolescent girls who exhibit neural sensitivity to *peer* rejection report more reactivity to perceived social threat during real-world peer interactions (Sequeira, Silk, et al., 2021) and exhibit affect-biased attention for social threat (e.g., a critical judge during a speech task; Sequeira, Rosen, et al., (2021).

This study had several notable strengths, including the use of multiple methods high in ecological validity. Specifically, interviewer-coded levels of expressed emotion criticism and intensive, high-quality, self-report data assessed via EMA were used to understand how adolescents with critical parents experience their day-to-day interactions with peers. Yet, limitations also warrant consideration. First, results suggest a significant positive association between parental criticism and adolescents’ perceptions of social threat in peer interactions; however, as previously discussed, the design of the current study limits our ability to determine directionality, including the extent to which effects are bi-directional. Next, we note several limitations related to our sample: Adolescents in this study primarily identified as white, and all were assigned female at birth. Similarly, almost all of the participating parents in the study identified as mothers. These sample characteristics limit the generalizability of our results to other demographic groups. Future studies with more diverse samples that include adolescents assigned male at birth and a larger number of fathers are needed to improve generalizability and examine potential sex-differences in these relations. Additionally, our sample size was relatively small, which may have limited power for some analyses (e.g., moderation models, as described above). Finally, one parent per family participated in the current study. Consequently, we only assessed levels of parental criticism from one parent. It is likely that some non-participating parents of adolescents in our study also exhibit high levels of criticism that were not captured in the present work. Given the design of the current study, we are unable to evaluate how many of the adolescents in our low criticism group have a critical non-participating parent. We are also unable to examine the potential effect of having more than one critical parent.

Finally, we note some limitations related to our measures. First, the current study focused on the presence of parental criticism as defined by the FMSS coding system rather than the specific content of the criticism (e.g., the behaviors/traits being criticism). Future research is needed to develop a more nuanced understanding of the relation between exposure to specific types of parental criticism and adolescents’ socio-affective functioning, including potential differences based on how broad versus specific parents’ criticism is and the types of behaviors criticized. Moreover, emotion socialization theories suggest that adolescent state and trait mood likely interact with parental criticism in dynamic and bidirectional ways to predict daily-life social experiences (Eisenberg, 2020; Eisenberg et al., 1998; Morris et al., 2007). Research is needed using advanced ambulatory assessment methods to evaluate fluctuating levels of parental criticism *in daily life* to disentangle these potential dynamic and bidirectional effects. Second, given our focus on ecological validity and challenges disentangling appraisal/bias from objective experiences of stress using checklists measures (as would be used in an EMA study; Harkness & Monroe, 2016; Rnic et al., 2023), the current study did not objectively measure adolescents’ day-to-day social stressors. Despite potential sacrifices in ecological validity, future research is needed to determine the extent to which adolescents with critical parents also experience higher levels of objectively threatening peer interactions, though it is likely that adolescents’ perception matters more than objective experiences (Jeon et al., 2013; Letkiewicz et al., 2023). Finally, participants were asked to report levels of perceived social threat/reward since the last assessment point (e.g., a few hours ago) rather than in the moment. Therefore, participants’ reports may have been vulnerable to some retrospective recall bias.

In sum, the current study suggests that exposure to a critical parent is associated with how adolescent girls experience day-to-day interactions with their peers. Adolescents exposed to a critical parent may be especially attuned to social threat during their peer interactions and/or to experience social stressors as more threatening than peers without a critical parent, potentially increasing their risk for STBs. Future longitudinal research that replicates and extends these findings could clarify potential mechanisms of risk by which parental criticism increases risk for STBs among adolescent girls, and provide specific, real-world behavioral targets for a new generation of targeted prevention and intervention efforts to prevent suicide deaths during adolescence and beyond.

## Supplementary Information

Below is the link to the electronic supplementary material.

Supplementary Material 1 (DOCX 21.0 KB)

## Data Availability

Data and code will be made publicly available through OSF once the manuscript has been accepted for publication.

## Footnotes

1 Due to the small number of participating fathers, sensitivity analyses were conducted including only maternal FMSS data. Results were consistent with findings generated using the full sample. Thus, all available data was included for the purposes of the present analyses in the interest of power. For further details regarding these sensitivity analyses, interested readers are encouraged to contact authors.

2 1% of youth preferred not to disclose their gender identity.

3 Multilevel correlations were calculated using the ‘misty’ package in R (Yanagida, 2024; R Core Team, 2024). Together, correlation coefficients and multilevel reliability estimates support the hypothesized one factor structure of the present measures of social threat and reward. Of note, the magnitude of within-persons correlation coefficients of some items are relatively smaller than others (although still medium in size). Rather than interpreting this to mean that one item measures a separate construct, we believe this provides evidence that our items capture a sufficiently wide breadth of a single construct.

4 Multilevel models including random slopes did not converge, indicating inferior model reliability relative to multilevel models including random intercepts alone. Additional three-level models were also tested to account for nesting of individual observations (level 1) within days (level 2) within people (level 3). Patterns of results were unchanged relative to two-level models. Thus, concerns for power and parsimony, results of two-level models are reported in the present manuscript. For further details regarding three-level models, interested readers are encouraged to contact study authors.

5 Fit statistics for all models are reported in Supplemental Table S2.

6 Additional sensitivity analyses were conducted including only participants who completed > 25% of EMA surveys. Results of these models are reported in Supplemental Table S3.
