# Supplementary material for: Parental Expressed Emotion Criticism Relates to Everyday Perceptions of Social Threat in Adolescents with Varying Suicidal Thoughts and Behaviors
Source: Res Child Adolesc Psychopathol. 2026 Feb 16;54(2):34. doi: 10.1007/s10802-025-01416-9 (PMC12909432; doi:10.1007/s10802-025-01416-9)
Supplement: Supplementary file 1 — Supplementary Material 1 (DOCX 21.0 KB) [file 10802_2025_1416_MOESM1_ESM.docx]

Table S1

*Results of Sensitivity Analyses Controlling for Relevant Covariates*

|  | *b* | SE(*b*) | *p* |
| --- | --- | --- | --- |
| **Parental Criticism 🡪 Perceived Social Threat** | | | |
| Intercept | 25.69 | 2.77 | <.001 |
| Parental criticism | 7.25 | 3.90 | .060 |
| Depressive symptoms | .45 | .26 | .089 |
| Anxiety symptoms | .18 | .12 | .142 |
| Suicidal ideation | .08 | .15 | .562 |
| Age | -1.82 | 1.21 | .137 |
| EMA completion rate | 2.08 | 11.41 | .856 |

*Note*. *b*=unstandardized effect; SE(*b*)=standard error of the unstandardized effect.

Table S2

*Fit Statistics for Multilevel Models*

| **Model** | **Log Likelihood (df)** | **AIC** | **BIC** |
| --- | --- | --- | --- |
| Parental Criticism 🡪 Perceived Social Threat | -7762.74 (4) | 15533.47 | 15555.57 |
| Parental Criticism 🡪 Perceived In-Person Social Threat | -5829.97 (4) | 11667.95 | 11688.82 |
| Parental Criticism 🡪 Perceived Digital Social Threat | -6276.18 (4) | 12560.36 | 12581.58 |
| Parental Criticism 🡪 Perceived Social Threat Controlling for Relevant Covariates | -6771.44 (9) | 13560.89 | 13609.48 |
| Parental Criticism 🡪 Perceived Social Reward | -8148.03 (4) | 16304.07 | 16326.18 |

*Note*. df=degrees of freedom; AIC=Akaike information criterion; BIC=Bayesian information criterion.

Table S3

*Results of Sensitivity Analyses Excluding Participants with Low Completion Rates (<25%)*

|  | *b* | SE(*b*) | *p* |
| --- | --- | --- | --- |
| **Parental Criticism 🡪 Perceived Social Threat** | | | |
| Intercept | 22.24 | 2.82 | <.001 |
| Parental criticism | 12.31 | 3.83 | .002 |
| **Parental Criticism 🡪 Perceived Social Reward** | | | |
| Intercept | 66.83 | 2.44 | <.001 |
| Parental criticism | -6.55 | 3.30 | .050 |

*Note*. *b*=unstandardized effect; SE(*b*)=standard error of the unstandardized effect.
